# Interannual Variation in Root Production in Grasslands Affected by Artificially Modified Amount of Rainfall

**DOI:** 10.1100/2012/805298

**Published:** 2012-05-02

**Authors:** Karel Fiala, Ivan Tůma, Petr Holub

**Affiliations:** ^1^Department of Vegetation Ecology, Institute of Botany, Academy of Sciences of the Czech Republic, Lidická 25, 602 00 Brno, Czech Republic; ^2^Department of Agrochemistry, Soil Science, Microbiology and Plant Nutrition, Faculty of Agronomy, Mendel University in Brno, Zemědělská 1, 613 00 Brno, Czech Republic; ^3^Global Change Research Centre, Academy of Sciences of the Czech Republic, Bělidla 4a, 603 00 Brno, Czech Republic

## Abstract

The effect of different amounts of rainfall on the below-ground plant biomass was studied in three grassland ecosystems. Responses of the lowland (dry *Festuca* grassland), highland (wet *Cirsium* grassland), and mountain (*Nardus* grassland) grasslands were studied during five years (2006–2010). A field experiment based on rainout shelters and gravity irrigation simulated three climate scenarios: rainfall reduced by 50% (dry), rainfall increased by 50% (wet), and the natural rainfall of the current growing season (ambient). The interannual variation in root increment and total below-ground biomass reflected the experimentally manipulated amount of precipitation and also the amount of current rainfall of individual years. The effect of year on these below-ground parameters was found significant in all studied grasslands. In comparison with dry *Festuca* grassland, better adapted to drought, submontane wet *Cirsium* grassland was more sensitive to the different water inputs forming rather lower amount of below-ground plant matter at reduced precipitation.

## 1. Introduction

Predicted scenarios of global change include an increase of drought during the growing season and higher frequencies of extreme rainfall events [1]. Their effects on root production of various grasslands are mostly unknown. Changes in the amounts and timing of rainfall events will probably affect ecosystem processes, including those that control carbon (C) cycling and storage. If temperature and rainfall conditions would change more rapidly than the change of CO_2_ concentration in the atmosphere, their consequences could be much more serious [2]. These climate changes may affect the supply of C and energy to the soil microbial populations and subsequently alter decomposition and mineralization processes. Seasonal variation in precipitation and temperature are important controls of soil and plant processes in grasslands. Such changes may affect numerous soil, plant, and ecosystem properties in grasslands and ultimately influence their productivity and biological diversity [3–5].

Root mass and rhizosphere represent the main pool of organic matter and geobioelements of grassland ecosystems [6–8]. As these ecosystems store up to 30% of the world below-ground C, it is important to understand how variability in climate factors affects soil C pools/fluxes, and how C cycling might be affected by changes in precipitation, due to climate change [9]. The relationships between rainfall and aboveground biomass production of grasslands have been studied quite frequently (e.g., [10–13]). The effect of water stress on grass growth and dry matter production mostly prevailed over other stress factors. The biomass of meadows mostly decreased with decreasing rainfall, reflecting so mainly the impairment of plant nutrition [14].

Production of new roots was often observed during periods of favourable soil water conditions and dry periods coincided with a decline of root dry mass (e.g., [15–20]). To the contrary, Bakker et al. [21] assessed that total fine root biomass and total fine root length were significantly higher at the dry site than at the humid site, in accordance with studies by Ibrahim et al. [22], Qaderi et al. [23], and Wedderburn et al. [24]. Some of them also mentioned that the significant decline in living roots and increase in dead roots corresponded with drought. Thus the total dry mass of below-ground plant parts comprises also dead undecomposed plant matter. Amounts of decomposed dead plant parts are associated, beside others, with differences in soil moisture of various ecosystems (e.g., [25–30]). Lower precipitation rate or soil moisture can mostly reduce plant matter decomposition.

Summary of the published results indicates that contradictory data on root growth and below-ground biomass in dry conditions were often presented. Although there are data on the interaction between changes in rainfall recorded over several years and the above-ground biomass production, a gap in knowledge still exists on the interannual variation in root growth and below-ground plant biomass accumulation in various grasslands at different water availabilities. Therefore, our main objective was to determine the effects of changes in rainfall amounts on the biomass production of roots. This was studied in three different grassland ecosystems occurring in lowland, highland, and mountain regions. Although rainout shelters were used by several authors [4, 31–33], the effects of different amounts of precipitation on interannual variation of root growth were not studied by them. We expected that combination of the data from an altitude gradient and from a moisture gradient will show how rainfall controls the root production and the biomass accumulation. For this goal we used data from five-year field experiments which combined naturally varying and artificially manipulated precipitation. Main and a new contribution of our study to the problem was that we obtained data with the help of two different and simultaneously used methods and results were gathered during a relatively long period of five years.

As smaller amounts of precipitation cause reductions in above-ground production, we expected to find lower root increments and lower biomass allocation to below-ground plant parts in drier years and in reduced rainfall treatments. We hypothesized that

1. experimentally manipulated amounts of rainfall control root growth in grasslands such that the lowest yearly root increments take place in the drier treatments,
2. the lowest accumulation of total below-ground plant parts occurs in reduced amounts of rainfall,
3. interannual variation in root production and accumulation are characterized by their lower values recorded in dry years.

## 2. Material and Methods

### 2.1. Study Sites

This study was conducted during five years (2006–2010) at three sites in different grassland ecosystems. They were situated in (1) a lowland site (the Podyjí National Park near the town of Znojmo, etchplain in the southern Moravian lowland—lowland grassland), (2) a highland (the Moravian-Bohemian Highland near the village of Kameničky, SE of the town of Hlinsko—highland grassland), and (3) a mountain region (near the locality Bílý Kříž in the Moravian-Silesian Beskydy Mts.—mountain grassland). The mean annual temperature and precipitation (for the period 1961–1990) in these regions ranged from 8.5°C and 471 mm (Znojmo, Kuchařovice), through 7°C and 762 mm (Kameničky, Svratouch), to 6.5°C and 947 mm (Bílý Kříž). A nutrient-poor shallow soil of the Ranker type occurred in the lowland site (the bedrock is formed by granite), a brown acid gleyed soil on crystalline rocks in the highland site, and a spodo-dystric cambisol (podzol brown soil) on Flysch Godulian sandstone in the mountain site (Table 1). The lowland grassland was located near the village of Havraníky (altitude 320 m). It was covered with dry acidophilous short grass vegetation dominated by the graminoids* Festuca ovina*, *Avenella flexuosa, Anthoxantum odoratum, Arrhenatherum elatius* and dicots *Pimpinella saxifraga, Potentilla arenaria, Trifolium arvense, Achillea millefolium*, and others. The highland grassland was characterized as a species rich stand of wet *Cirsium* meadow (altitude 530 m). The species composition of the stand was characterized by frequent species such as *Cirsium palustre, Deschampsia ceaspitosa, Agrostis capilaris, A. canina, Anthoxanthum odoratum, Polygonum bistorta, Sanguisorba officinalis*, and others. A mountain *Nardus *grassland occurred on the third site in the Beskydy Mts. (altitude 890 m). Its main components are *Nardus stricta, Avenella flexuosa, Festuca rubra,* and* Agrostis capillaris. *The monocots *Holcus mollis *and *Carex pilulifera* and dicots *Veronica officinalis* and *Hieracium laevigatum* are also frequently present. All stands were not cut in the course of the experiment.

**Figure 1:**
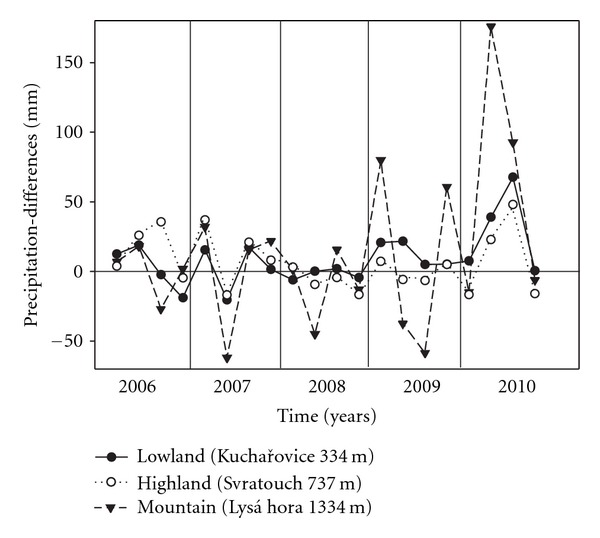
Differences in the amount of precipitation from the long-term average values (1961–1990) recorded at the meteorological stations Kuchařovice (lowland), Svratouch (highland), and Lysá hora (mountain, approximately 9, 4, and 8 km from studied sites, resp.) and calculated for quarters of the years of 2006 to 2010.

The amount of rainfall (ambient treatment) recorded during the growing seasons at the studied sites fluctuated between 339 (recorded in 2007) and 606 mm (in 2010) in the lowland site and between 426 (in 2008) and 794 mm (in 2010) in highland site (Table 2). At the mountain site, the precipitation showed less variation over the years (532–601 mm), but a considerable fluctuation within years. This was caused mostly by storm rainfalls (810 mm recorded in 2007). A comparison of the deviations of the amount of actual precipitation from the long-term average values (1961–1990) recorded at nearby meteorological stations and calculated for quarters of the individual years (2006 to 2010) indicates that the stands received a lower amount of precipitation at the beginning of the growing seasons 2007 and 2008 (second quarters, Figure 1). The data on the amount of precipitation measured at the studied sites and comparison with data from meteorological stations indicate that the 2007 and 2008 growing seasons were drier particularly in comparison with 2006 and 2010 (Figure 1). In the highland grassland, soil moisture conditions given by amount of precipitation occurring from the end of May to June were also influenced here by a higher underground water table, ranging about −20 cm below soil surface due to the melting of a huge amount of snow. However, in summer months, underground water table decreased here often down to −50 cm.

### 2.2. Experimental Design

Twelve 2 × 3 m plots were laid out in an area of relatively homogeneous grasslands at each of the three localities (four replications of each treatment were in block design). Rainout shelters constructed above the canopy of grass stands and a gravity irrigation system simulated three scenarios: (1) rainfall reduced by 50% (dry treatment), (2) rainfall enhanced by 50% (wet treatment), and (3) the full natural rainfall of the current growing season (ambient treatment—amb). For the dry treatment, rainout shelters constructed over the experimental plots consisted of a steel frame supporting plastic transparent strips (small troughs, see [34]) that covered 50% of the experimental plots. Such rain water shelters with a roof consisting of bands of transparent blocks represent well-replicated experiments with minimal secondary microenvironmental effects [34]. The water was piped as gravity irrigation into the corresponding wet treatment plots. A 0.2 m wide trench was dug and sheathed with a plastic foil to separate the soil of the roofed and irrigated areas from the neighbouring soil. No measurements were performed in a 0.25 m wide peripheral.

### 2.3. Below-Ground Plant Parts Analyses

In order to assess yearly root increments (root production), the in-growth core technique was used during five years. Eight plastic-mesh tubes with river sand were inserted into holes (5 cm in diameter, 15 cm depth) in experimental treatments (two in each replicated plot) at the beginning of the growing season. The tubes were lifted at the end of the growing season and roots were washed, dried, and weighed. The total below-ground biomass (TBB) was collected at the end of five growing seasons. Eight soil cores were taken to the depth of 15 cm in experimental plots with a root auger (diameter 9.4 cm) representing more than 90% of total below-ground dry mass of studied plant communities. The below-ground plant parts were washed free of soil over a 0.5 mm mesh sieve. Samples were separated into total roots and rhizomes with shoot bases (referred as rhizomes for simplification), dried, and weighed.

### 2.4. Statistical Analysis

Data were evaluated by an analysis of variance, using statistical package STATISTICA 9. A repeated measures ANOVA analysis was used to test the effect of manipulated rainfall as nonrepeated factor on both root increments and dry mass of below-ground plant parts, where the years were used as repeated measures factor. Significant differences among means were tested (Tukey HSD test (*P* < 0.05) after ANOVA). Linear correlation analyses were performed between root increments, TBB, and precipitation input to determine whether these parameters are related.

## 3. Results

### 3.1. Yearly Root Increments and Their Interannual Variations

**Figure 2:**
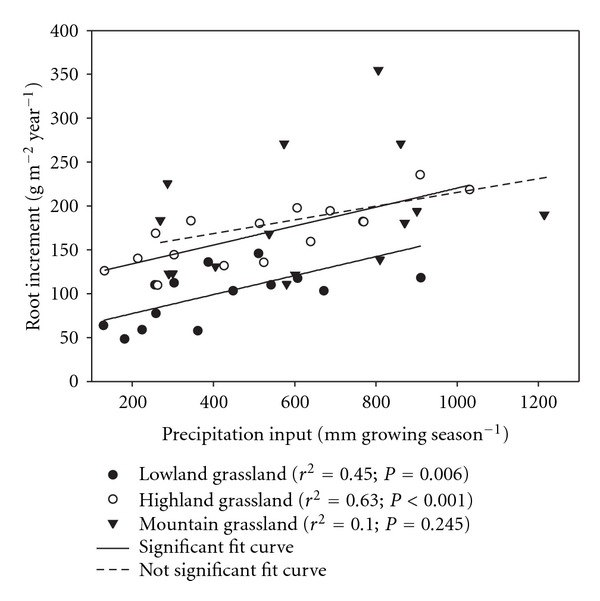
Relationship between the yearly root increment and precipitation input along the experimental precipitation gradient. Each point indicates annual mean.

**Figure 3:**
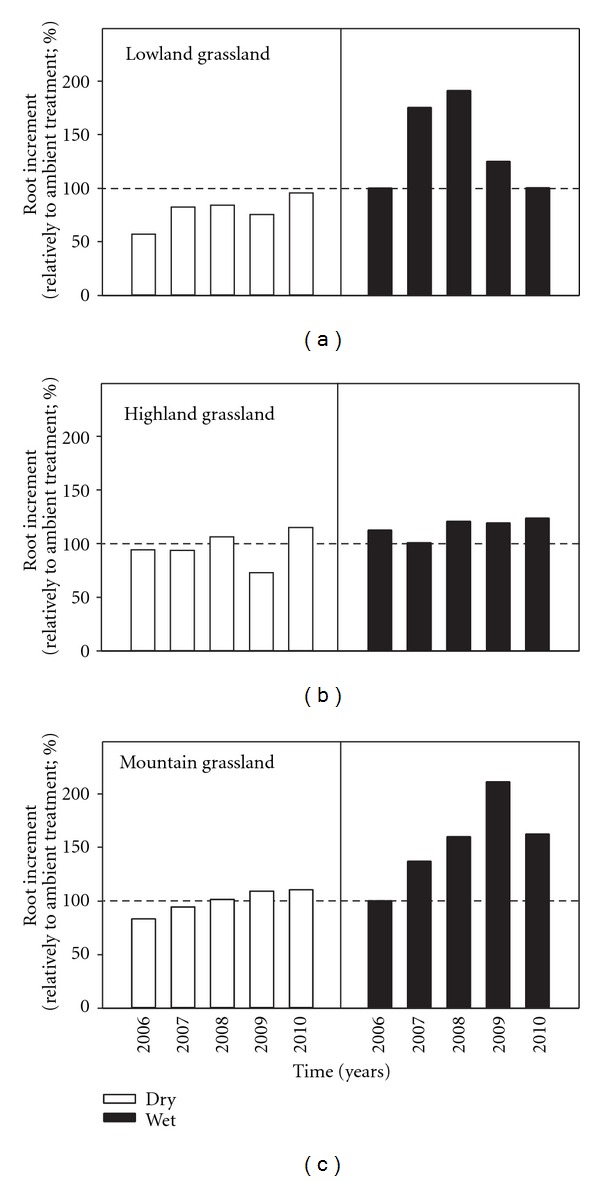
Percentage increase or decrease in yearly root increment (ambient treatment = 100%) in dry and wet treatments recorded in five years (2006–2010).

The repeated measures analysis has shown that the yearly root increments (YRIs) were significantly affected by rainfall input in the lowland and mountain grasslands (Table 3). In addition, correlation analysis summarizing five-year data indicated that the YRI increased with enhanced rainfall input, but significantly only in lowland and highland grasslands (Figure 2). Thus the effect of rainfall input on root increment was observed in all studied grasslands. In the highland site, the rather variable data on YRI recorded during five years may explain the mostly nonsignificant results. Although often not significant, a tendency to a higher YRI was found in wet treatments compared to the dry treatments in all studied grassland (Table 4). The percentage increase or decrease in YRI in dry and wet treatments in comparison with the ambient precipitation recorded in five years (2006–2010) is summarized in Figure 3. According to 5-year means, marked increase in root production (38, 15, and 54%) has been observed in the wet treatment in the lowland, highland and mountain grassland, respectively. On the other hand, 21% reduction (in average) of root production was found in dry treatment of the lowland grassland, while no effect of decreased rainfall input was noted in the highland and mountain grasslands in comparison with ambient treatment (Figure 3).

In the first three years (2006–2008), the YRI recorded in the dry treatment of the lowland *Festuca* grassland represented, on the average, only about 49% (57 g m^−2^ year^−1^) of the root biomass formed in stands affected by higher precipitation input (wet treatment, Table 4). However, in 2009 and 2010, a significantly greater root production (mostly above 100 g m^−2^ year^−1^) was formed in *Festuca* grassland in the dry treatment than in previous years and even reached values recorded in the wet treatments in 2010 (Table 4). In the course of five years, a decreasing tendency in YRI was found with time in all rainfall input treatments of the highland *Cirsium* grassland. This was documented by the highest significant values recorded in 2006 (183 and 219 g m^−2^ year^−1^ in the dry and wet treatments, resp.) and lower amount of roots recorded in 2010 (69, resp., 63% of the values at the beginning of experiment, Table 4). Substantial increases of YRI were found in the ambient and wet treatments only in 2009, probably due to improved water conditions after two relatively dry years. In mountain *Nardus* grassland, mostly significantly higher values of YRI were assessed through all treatments in the 2006 and 2009 growing seasons (Table 4). During five years, the data on YRI averaged here 156, 140, and 229 g m^−2^ year^−1^ in the dry, ambient, and wet treatments, respectively. The significantly highest YRI value (355 g m^−2^ year^−1^) was found in the wet treatment in 2009.

### 3.2. Below-Ground Plant Parts and Their Interannual Variations

**Figure 4:**
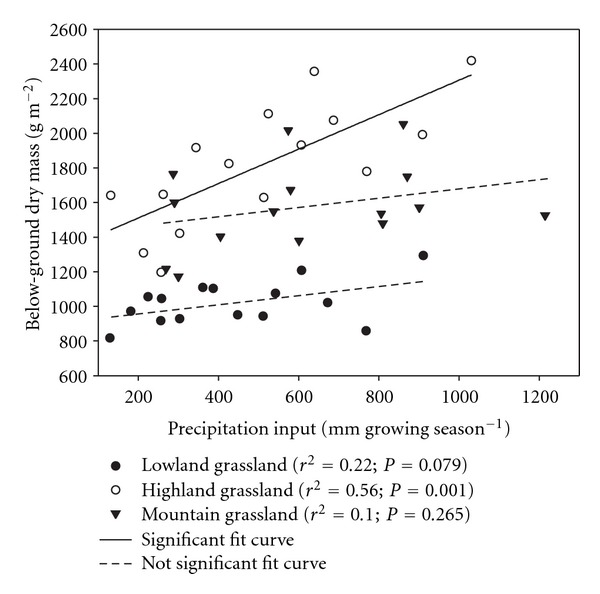
Relationship between the amount of total below-ground plant dry mass and precipitation input along the experimental precipitation gradient. Each point indicates annual mean.

The repeated measures analysis confirmed the significant effect of a varying rainfall input on the accumulation of roots, rhizomes, and TBB in highland and rhizomes in lowland grasslands (Table 3). Concerning interactions, below-ground plant parts were not affected by the interaction of rainfall input and year, except rhizomes in lowland grassland. In addition, the increasing dry mass of both roots (not presented) and TBB in the stand of highland *Cirsium* grassland correlated positively with the increasing amount of precipitation (Figure 4). Values recorded in the other two grassland types were more variable and this relationship was not significant (Figure 4). The greatest differences in below-ground plant parts were usually recorded between dry and wet treatments. In lowland grassland, TBB fluctuated in a narrow range of values and differences between plant parts and years were mostly not significant. Nevertheless, these changes have shown here significant differences between dry and wet treatments recorded in the second year of experiment (Table 5). Similar significant decreases were also found in mountain grassland, but only for roots (2008 and 2009) and TBB (2008). In the highland grassland, however, significant differences in below-ground plant parts between dry and wet treatments were found nearly in all years (Table 5).

**Figure 5:**
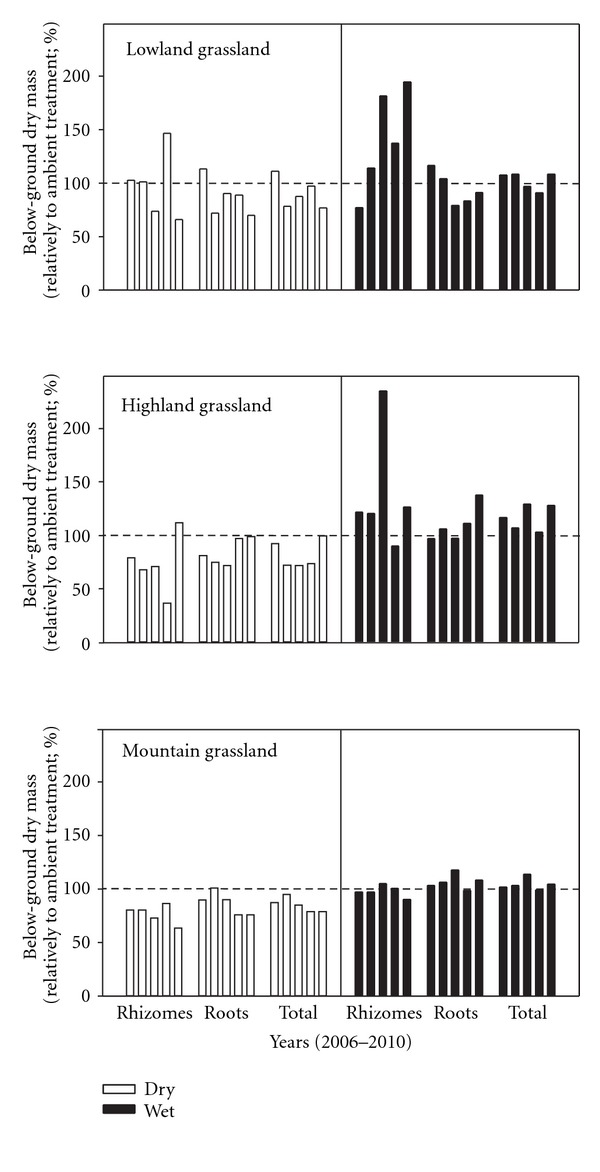
Percentage increase or decrease in dry mass of rhizomes, roots, and total below-ground plant biomass (ambient treatment = 100%) in dry and wet treatments recorded in five years (2006–2010).

During five years studied, the TBB fluctuated between 1197 and 1916 g m^−2^ in dry and 1779 and 2419 g m^−2^ in wet treatments of the highland grassland. In addition, the TBB ranged between 818 and 1056 g m^−2^ in dry and 859 to 1294 g m^−2^ in wet treatments in the lowland grassland and between 1172 and 1766 g m^−2^ in dry and 1526 to 2054 in wet mountain grassland (Table 5). Thus TBB of those grasslands was generally lower in comparison with values of *Cirsium* highland grassland. According to 5-year means, both the greatest reduction (by 15 and 18%, resp.) and accumulation (10 and 17%, resp.) of the root biomass and TBB have been noted in the highland grassland (Figure 5). On the other hand, the mountain grassland was characterized by relatively stable variations in the amount of below-ground plant parts under different amounts of rainfall (Figure 5). In addition, the decrease in the amount of rainfall input resulted also in a lower amount of rhizomes in dry treatment than that in wet treatments in highland (significant in 2007–2009) and lowland grasslands (significant in 2008) (Table 5).

Interannual changes in below-ground biomass of lowland *Festuca* grassland were characterized by fluctuation of data in a narrow range of values and differences between them were mostly not significant (Table 5). Nevertheless, several significant differences in roots and TBB between dry and wet treatments were found in the second year (2007). In highland and mountain grasslands, respectively, a considerable significant reduction of roots (by 327 and 565 g m^−2^) and TBB (by 445 and 539 g m^−2^) occurred in ambient treatments in the second year (2007) in comparison with the previous year. A decreasing tendency in the dry mass of these plant parts also occurred in the following two years, particularly in the dry treatment of the mountain grassland (Table 5). The greatest significant differences between rainfall input treatments were here found in the third year when 1571 and only 1172 g m^−2^ of TBB accumulated in wet and dry treatments, respectively. On the contrary, an increase in root and TBB mostly occurred in all treatments in the studied grasslands in the last year (2010). In all grasslands studied, the mean values of dry mass of rhizomes including shoot bases were also lower in the dry in comparison with wet treatments, but mostly not significantly (Table 5). In the highland grassland, however, the pronounced reduction of rhizomes recorded due to lower precipitation was mostly significant.

## 4. Discussion

### 4.1. Yearly Root Increments and Their Interannual Variations

Our assumption that root growth is affected by experimentally manipulated rainfall inputs was confirmed for all studied grasslands. However, this fact was documented by significant effects of rainfall input treatments in ANOVA analyses in lowland and mountain grasslands and by correlation analyses which demonstrated that the yearly root increment (YRI) increased linearly with increasing precipitation in lowland and highland grasslands.

Our results are supported by findings of several authors. For example, the lowest yearly production of new roots and root elongation rates were found due to a decrease of the soil water content (e.g., [17, 35, 36]). Based on a large collection of field measures, Hui and Jackson [8] concluded that the proportion of the below-ground net biomass production in the total net primary production was negatively correlated with the average annual temperature and precipitation across sites. Our results indicate higher root increments in cool and wet highland and mountain sites, while lower values in dry and warmer environments of lowland sites were found. Perez and Frangi [37] reported that below-ground net productivity in grassland sites increased with altitude. On the other hand, a greater root production at lower than at higher elevated sites was found in several temperate grasslands [38]. Nevertheless, root production may not be a simple function of altitude [38, 39]. In our case, both altitude and amount of rainfall can explain obtained results of individual sites.

The repeated measures analysis also exhibited the effect of year on YRI in all studied grasslands. The results show the lowest YRI in the dry treatment of the lowland dry grassland and a decreased root production in the dry treatments of the highland and mountain grasslands, particularly during the first three years. This fact can be associated with the lower regular rainfall recorded during this period. The YRI significantly increased in the *Festuca* lowland grassland in 2009 and 2010. In these years, the amount of precipitation was above the long-term averages. In the mountain grassland, the YRI varied over a wide range of values. This could also be associated with fluctuating amounts of the current precipitation. Production of new roots was observed during periods of favourable soil water conditions [40, 41] and the decline in the below-ground net biomass production was found in dry years [15, 16, 18]. In addition, the below-ground net primary production was not related to the early but to the late rainfall in the rainy season [17]. However, Fitter et al. [39] concluded that a yearly increase in root biomass can be rather a function of changes in length of the growing season, not soil temperature. In the present study, in the drier vegetation seasons (2007 and 2008) decreased YRIs were recorded in nearly all grasslands and treatments, but particularly in the mountain grassland. Thus these interannual variations in root production reflected not only the experimentally manipulated amount of precipitation but also the current rainfall, that is, dry and wet conditions of individual growing seasons.

### 4.2. Below-Ground Plant Parts and Their Interannual Variations

We expected to find a lower accumulation of below-ground plant parts in dry conditions. The repeated measures analysis showed that data of dry mass of all below-ground plant parts of the studied wet *Cirsium* grassland in highland were only significantly affected by the rainfall input treatment. In this grassland, the introduced reduction and increase of amounts of rain were connected with a decrease or increase in all below-ground plant parts. Correlation analysis suggested a significant positive relationship between precipitation input and root and TBB in the highland grassland. These our results support the hypothesis that the different amounts of rainfall will be reflected in the below-ground plant biomass and the lowest accumulation of total below-ground plant parts will occur in reduced amounts of rainfall.

Dry conditions appear to influence the root mortality (e.g., [24, 40–42]). Above all summer droughts can lead to increased root mortality, thereby reducing root biomass. Hayes and Seastedt [15] also mention that the significant decline in living roots and increase in dead roots corresponded with drought. Therefore the disappearance of roots and consequently decrease in root dry mass could have resulted from the low rainfall. Decomposition processes can modify the amount of TBB in different soil moisture conditions, resulting in varying accumulation of below-ground undecomposed plant litter. Drought mostly resulted in a decline of below-ground dry mass [43].

The repeated measures analysis also showed that TBB of all studied grasslands changed significantly with year. Thus both experimentally and naturally altered rainfall inputs were associated with variation in values of the below-ground dry mass, although not significantly in all five years. Not significant data on interactions between rainfall input and year indicated that dry or wet years reduced or increased below-ground biomass in dry and wet treatments in the same extent. The interannual variation was characterized by a decreasing tendency in the amount of TBB after experimental reduction of precipitation in all studied grasslands. In addition, a considerable reduction of the TBB occurred through all rainfall input treatments in the studied highland and mountain grasslands in the second year of the experiment (2007). In comparison with other years, the grasslands received the lowest amount of precipitation in the first part of this growing season. In wet *Cirsium* highland grassland, differences in water availability were reflected, mostly significantly, in accumulated root dry matter. Therefore, our last assumption to find a lower root accumulation in dry years was confirmed in wet *Cirsium* grassland.

The results of the present study correspond with data of other authors (e.g., [15, 18, 44, 45]) who noted that root biomass and root length were lower in dry years. Drought and soil moisture decrease reduced decomposition processes of dead plant matter, whereas enhanced soil moisture can accelerate decomposition below-ground in many ecosystems (e.g., [26, 27, 29, 30, 46]). Changes in the distribution of the rain during the year may be more important than changes in the total amount of rain. Mid-growing season drought can result in accelerated death and decomposition of new roots [15]. Below-ground turnover rates in grasslands decreased with altitude [37]. This fact was confirmed by data on total root dry mass from different regions summarized by Fiala [47, 48]. Therefore the total dry mass of below-ground plant parts comprises also various amounts of dead undecomposed plant matter, in upper elevation sites particularly. Nevertheless, recorded increase of root biomass in studied years can indicate rather new root growth than the decreased decomposition.

## 5. Conclusions

Our results indicate the strong effect of reduced precipitation on decrease of roots and TBB in the wet submontane *Cirsium *grassland, occurring often in the central European region. Below-ground plant matter is considered as stabilizing element of grasslands, which also functioning as water storage in the landscape. Therefore a substantial reduction of root matter can contribute to destabilization of grassland ecosystems. In addition, the wet submontane *Cirsium *grassland often occurs in spring regions providing supply of drinking water. Although the YRI decreased linearly with decreasing precipitation in lowland and highland grasslands, the same relationship was not found for roots and TBB in the mountain. In the studied mountain *Nardus* grassland, amount of precipitations cannot be always the main predictor of the amount of below-ground plant biomass due to relatively high current rainfall. The dry *Festuca* grassland can be better adapted to dry conditions and below-ground biomass fluctuated here in a narrow range of values. The new information related to the influence of precipitation on growth and accumulation of roots is particularly important, because most current literature has focused on the aboveground biomass production, but grasslands accumulate larger plant biomass below-ground.

## Figures and Tables

**Table 1:** Soil features of grass stands of studied sites (soil layer 0–10 cm).

Features	Lowland site	Highland site	Mountain site
pH-H_2_O	5.4	5.1	4.7
pH-KCl	4.6	4.3	3.8
Organic matter (%)	9.0	13.9	8.6
P (mg kg^−1^)	44.7	23.0	4.0
K (mg kg^−1^)	359	167	140
Mg (mg kg^−1^)	166	163	57
Ca (mg kg^−1^)	1249	1589	426
N tot (%)	0.34	0.44	0.39

**Table 2:** Amount of precipitation (the full natural rainfall: ambient treatment in mm per growing season) recorded at the studied sites (Lowland: Havraníky, Highland: Kameničky; Mountain: Bílý Kříž) in the growing seasons 2006 to 2010.

Year	Lowland grassland	Highland grassland	Mountain grassland
2006	446	687	574
2007	339	513	810
2008	361	426	601
2009	511	606	532
2010	606	794	580

**Table 3:** The effect of rainfall input and year on the root increment, roots, rhizomes, and total below-ground biomass (TBB): results of repeated measures analysis of variance (ANOVA), using years as repeated measures factor (NS: not significant, *P < 0.05, **P < 0.01, ***P < 0.001; df error = 84).

Effect		Root increment		Rhizomes		Roots		TBB	
	df	* F*	*P*	*F*	*P*	* F*	*P*	* F*	*P*
Lowland grassland									

Rainfall input	2	8.0	**	4.5	*	2.0	NS	2.4	NS
Year	4	12.4	***	0.9	NS	1.9	NS	2.6	*
Interaction	8	1.5	NS	2.1	*	1.8	NS	1.2	NS

Highland grassland									

Rainfall input	2	2.5	NS	7.8	**	12.9	***	14.5	***
Year	4	8.8	***	1.7	NS	10.8	***	4.5	**
Interaction	8	1.0	NS	1.6	NS	1.3	NS	0.8	NS

Mountain grassland									

Rainfall input	2	9.3	**	1.2	NS	2.7	NS	5.4	NS
Year	4	11.0	***	3.2	*	19.7	***	10.8	***
Interaction	8	1.6	NS	0.1	NS	0.7	NS	0.4	NS

**Table 4:** Mean values (±SE) of yearly root increment under different amounts of precipitation (dry, ambient, and wet treatments) recorded in five years (2006–2010): results of one-way ANOVA analysis (NS: not significant, *P < 0.05, **P < 0.01, ***P < 0.001). Different letters denote significantly different values for rhizomes, roots, and total separately (Tukey HSD test (*P* < 0.05) after ANOVA).

Year	Yearly root increment			
	Dry	Ambient	Wet	*P*
Lowland grassland				

2006	58.8 ± 15a	103.1 ± 28b	103.2 ± 25b	**
2007	63.8 ± 16a	77.5 ± 17a	136.0 ± 63b	*
2008	48.5 ± 9a	57.6 ± 20a	110.1 ± 26b	***
2009	110.0 ± 19a	145.7 ± 43ab	182.4 ± 59b	NS
2010	112.4 ± 30a	117.6 ± 39a	118.1 ± 40a	NS

Highland grassland				

2006	182.9 ± 38a	194.4 ± 41a	218.6 ± 29a	NS
2007	168.6 ± 54a	180.1 ± 53a	181.8 ± 44a	NS
2008	140.2 ± 30a	131.9 ± 56a	159.4 ± 32a	NS
2009	144.2 ± 46a	197.8 ± 50ab	235.6 ± 50b	*
2010	126.0 ± 44a	109.8 ± 29a	135.8 ± 49a	NS

Mountain grassland				

2006	225.8 ± 69a	270.9 ± 44a	271.0 ± 73a	NS
2007	131.0 ± 28a	138.9 ± 75a	190.0 ± 83a	NS
2008	123.1 ± 59a	121.4 ± 41a	194.4 ± 47b	*
2009	183.5 ± 100a	168.0 ± 45a	354.8 ± 155b	*
2010	122.6 ± 34a	111.0 ± 31a	180.5 ± 23b	**

**Table 5:** Mean values (±SE) of dry mass of rhizomes, roots, and total below-ground biomass (TBB) under different amounts of precipitation (dry, ambient and wet treatments) recorded in five years (2006–2010): results of one-way ANOVA analysis (NS: not significant, *P < 0.05, **P < 0.01, ***P < 0.001). Different letters denote significantly different values for rhizomes, roots and total separately (Tukey HSD test (*P* < 0.05) after ANOVA).

Year	Rhizomes				Roots				TBB			
	Dry	Amb	Wet	*P*	Dry	Amb	Wet	*P*	Dry	Amb	Wet	*P*
Lowland grassland												

2006	225 ± 33a	219 ± 26a	169 ± 26a	NS	830 ± 34a	732 ± 52a	854 ± 81a	NS	1056 ± 56a	951 ± 56a	1022 ± 99a	NS
2007	165 ± 31a	163 ± 23a	186 ± 35a	NS	654 ± 39a	881 ± 75b	919 ± 53b	**	818 ± 47a	1045 ± 86b	1104 ± 72b	*
2008	142 ± 25a	192 ± 44a	349 ± 62b	*	829 ± 54a	917 ± 112a	764 ± 41a	NS	971 ± 40a	1109 ± 118a	1076 ± 72a	NS
2009	192 ± 26a	131 ± 29a	180 ± 41a	NS	725 ± 71a	813 ± 68a	679 ± 67a	NS	917 ± 74a	943 ± 71a	859 ± 94a	NS
2010	109 ± 20a	166 ± 25a	321 ± 122a	NS	729 ± 115a	1043 ± 103a	953 ± 154a	NS	928 ± 109a	1208 ± 118a	1294 ± 205a	NS

Highland grassland												

2006	367 ± 57a	464 ± 107a	565 ± 82a	NS	1549 ± 96a	1609 ± 72ab	1854 ± 94b	*	1916 ± 124a	2074 ± 171ab	2419 ± 142b	*
2007	236 ± 44a	347 ± 48ab	419 ± 72b	*	961 ± 99a	1282 ± 117b	1360 ± 100b	*	1197 ± 124a	1629 ± 149b	1779 ± 135b	*
2008	300 ± 26a	421 ± 98a	989 ± 310b	*	1009 ± 124a	1403 ± 56b	1368 ± 104b	*	1309 ± 121a	1824 ± 125b	2357 ± 294b	**
2009	276 ± 55a	754 ± 159b	680 ± 166b	*	1146 ± 131a	1179 ± 86a	1313 ± 129a	NS	1422 ± 180a	1932 ± 158ab	1993 ± 205b	*
2010	503 ± 148a	449 ± 138a	568 ± 108a	NS	1185 ± 104a	1198 ± 90a	1651 ± 118b	**	1641 ± 215a	1647 ± 194a	2112 ± 218a	NS

Mountain grassland												

2006	401 ± 102a	499 ± 82a	486 ± 76a	NS	1365 ± 116a	1519 ± 111a	1568 ± 211a	NS	1766 ± 151a	2018 ± 94a	2054 ± 184a	NS
2007	422 ± 114a	526 ± 76a	511 ± 92a	NS	982 ± 75a	954 ± 70a	1015 ± 125a	NS	1404 ± 103a	1479 ± 112a	1526 ± 113a	NS
2008	317 ± 97a	434 ± 78a	457 ± 68a	NS	855 ± 82a	947 ± 61ab	1114 ± 56b	*	1172 ± 96a	1380 ± 134ab	1571 ± 111b	*
2009	330 ± 77a	381 ± 92a	383 ± 94a	NS	888 ± 52a	1167 ± 49b	1154 ± 76b	**	1218 ± 105a	1548 ± 121a	1536 ± 157a	NS
2010	227 ± 48a	356 ± 61a	322 ± 55a	NS	1372 ± 91a	1318 ± 90a	1429 ± 53a	NS	1599 ± 130a	1674 ± 141a	1751 ± 48a	NS
